# Genetic characterization of a novel triple-reassortant influenza A (H1N2) virus from pigs, China, 2021

**DOI:** 10.3389/fmicb.2026.1779293

**Published:** 2026-04-09

**Authors:** Yuzhong Zhao, Chang Liu, Chenlu Xia, Yingchao Li, Yu Wang, Bingyu Hou, Hongyan Gao, Zhong Liu, Xiaotong Wu, Man Lu, Yang Shen, Pingping Yang, Yihong Xiao, Hongjie Yuan, Yanmeng Hou

**Affiliations:** 1 College of Veterinary Medicine, Shandong Agricultural University, Tai’an, Shandong, China; 2 Shandong Provincial Key Laboratory of Animal Biotechnology and Disease Control and Prevention, College of Veterinary Medicine, Shandong Agricultural University, Tai’an, Shandong, China

**Keywords:** genotype, H1N2, pathogenicity, reassortant, swine influenza virus

## Abstract

Swine influenza virus (SIV) is a highly contagious respiratory pathogen in pigs, with bidirectional transmission posing a potential threat to human health. In this study, nasal swab samples were collected from pigs in Shandong Province, China, and yielded an H1N2 SIV strain, designated A/swine/Shandong/QD726/2021 (H1N2). Whole-genome sequencing was performed for Sw/SD/QD726/2021, and phylogenetic analysis was conducted together with 156 Chinese H1N2 reference sequences obtained from the Global Initiative on Sharing All Influenza Data (GISAID) database and the National Center for Biotechnology Information (NCBI) Influenza Virus Resource database. The results indicated that Sw/QD726/2021 represents a novel reassortant genotype (G21), with the HA gene derived from Eurasian avian-like H1N1 (EA H1N1), the NA and NS genes from triple-reassortant H1N2 (TR H1N2), and the remaining internal genes (PB2, PB1, PA, NP, M) from the 2009 pandemic H1N1 (pdm/09 H1N1). Key amino acid analysis revealed N31 in M2, responsible for adamantane resistance, and S42 in NS1, which influences viral virulence in mouse models. BALB/c mouse experiments demonstrated efficient viral replication in the lungs and nasal turbinates, accompanied by moderate body weight loss and lung lesions, indicating only moderate pathogenicity. These findings underscore the ongoing evolution of H1N2 SIV in pigs and emphasize the importance of enhanced surveillance and preventive strategies to mitigate public health risks.

## 1 Introduction

Swine influenza is a highly contagious respiratory disease in pigs caused by the SIV (Joshi et al., 2021). Infected pigs often show symptoms such as coughing, sneezing, nasal discharge, fever, lethargy, and loss of appetite (Sun et al., 2021). SIV infection reduces growth and production performance and weakens the pigs’ immune system, making them more vulnerable to other infections. In addition, pig respiratory cells have receptors for both human and avian influenza viruses, which allows pigs to be infected by different strains. This makes pigs a “mixing vessel” for influenza viruses (Iwatsuki-Horimoto et al., 2017; Bourret et al., 2017), emphasizing the importance of studying swine influenza for public health.

Currently, the globally prevalent SIV primarily comprises three subtypes: H1N1, H1N2, and H3N2 (Trebbien et al., 2013). In 1978, Japan first isolated the H1N2 influenza virus from swine populations (Song et al., 2016), and this subtype was subsequently detected in regions including Asia, Europe, and North America (Yu et al., 2009). In April 2009, the pdm/09 H1N1 erupted globally and rapidly spread to swine populations. There, it underwent genetic reassortment with pre-existing SIV, giving rise to multiple H1N2 virus strains containing pdm/09 H1N1 gene fragments (Starick et al., 2012; Kanehira et al., 2014; Lange et al., 2013; Charoenvisal et al., 2013; Ali et al., 2012). Furthermore, certain H1N2 virus strains have caused human infections (Yang et al., 2022).

To understand the circulating SIV strains in pig herds in Shandong Province, China, nasal swab samples were collected from slaughterhouses, and an H1N2 SIV strain was successfully isolated. First, full-genome sequencing was carried out, and together with 156 Chinese H1N2 sequences downloaded from the GISAID and GenBank databases, a phylogenetic analysis was performed. The genotype of the strain was identified, and the key amino acid sites were examined to reveal its molecular features. Finally, a BALB/c mouse infection model was used to assess its pathogenicity. This study adds new data to the molecular epidemiology of SIV in China and provides a reference for the prevention and control of animal influenza.

## 2 Materials and methods

### 2.1 Ethics statement

All animal protocols and experiments were approved by the Animal Ethical and Experimental Committee of Shandong Agricultural University (Shandong, China). Well-trained and skilled animal care personnel participated in the current study to minimize the suffering of animals.

### 2.2 Sample collection and virus isolation

In December 2021, a total of 160 nasal swab samples were collected from pigs at a slaughterhouse in Shandong Province. The samples were immediately placed in transport medium containing penicillin (2,000 IU/mL), streptomycin (2,000 IU/mL), and 10% (v/v) glycerol, prepared in sterile phosphate-buffered saline (PBS, pH 7.2). Samples were transported to the laboratory at 4 °C and were stored at −80 °C until further processing.

An aliquot of 100 μL of each sample suspension was inoculated into the allantoic cavity of 9-day-old specific pathogen-free (SPF) embryonated chicken eggs using a sterile syringe. The eggs were incubated at 37 °C with 60–70% relative humidity, and embryo viability was monitored daily. Embryos that died within 24 h post-inoculation were considered nonspecific deaths and were excluded. At 48 h post-inoculation, allantoic fluids from viable embryos were collected and clarified by centrifugation at 3,000 × g for 10 min. The supernatants were then subjected to HA and RT-PCR assays to confirm the presence of SIV. Once confirmed, the supernatants were aliquoted and were stored at −80 °C for further analysis.

### 2.3 RT-PCR and sequence analysis

Viral RNA was extracted from 200 μL of allantoic fluid using TRIzol reagent (Invitrogen, United States) according to the manufacturer’s instructions. Complementary DNA (cDNA) was synthesized using the universal primer Uni12 (5′-AGC RAA AGC AGG-3′). The cDNA was then used as a template for PCR amplification of all eight influenza virus gene segments with universal primers (Hoffmann et al., 2001). PCR products were purified using a PCR purification kit (TianGen Biotech, Beijing, China) and were sequenced by Sanger sequencing at Shenggong Biotech (Shanghai, China).

The obtained sequences were assembled and edited using Lasergene software (DNAStar, Madison, WI, United States). Reference sequences were obtained from the GISAID database^1^ and NCBI’s Influenza Virus Resource database^2^ (accessed January 2025). Phylogenetic trees for all eight gene segments were constructed using the neighbor-joining method in MEGA 7.0. Branch support was assessed with 1,000 bootstrap replicates, and key branches with bootstrap values ≥70% were considered reliable for lineage assignment. Amino acid sequence alignments were also performed using MEGA 7.0.

### 2.4 Genotype classification

To classify the genotypes of H1N2 SIV isolates, a genotype was defined based on the combination of all eight gene segments (PB2, PB1, PA, HA, NP, NA, M, and NS) (Sun et al., 2025). Each unique combination of segment lineages was assigned a genotype number (G1–G28). The lineage of each gene segment was determined from phylogenetic analysis with reference sequences obtained from the GISAID and NCBI Influenza Virus Resource databases. This framework allowed identification of novel genotypes, such as G21, and comparison with previously reported genotypes to determine unique genetic features.

### 2.5 Determination of the 50% egg infectious dose (EID_50_)

The purified virus stock was serially diluted tenfold (10^−1^ to 10^−10^) using sterile PBS containing penicillin and streptomycin. 9-day-old SPF embryonated chicken eggs were used for inoculation, with five eggs per dilution. Each egg was inoculated with 100 μL of the corresponding viral dilution into the allantoic cavity. The eggs were then incubated at 37 °C for 48 h. After incubation, allantoic fluids were harvested and tested for HA activity. The number of HA-positive eggs at each dilution was recorded, and the EID₅₀ was calculated using the Reed–Muench method.

### 2.6 Mouse infection experiment

Six-week-old BALB/c mice (*n* = 16) were purchased from Pengyue Laboratory Animal Co., Ltd., Jinan, China. After 1 week of acclimation in standard cages, mice were randomly assigned to the control group (*n* = 8) or the infected group (*n* = 8). Each mouse was labeled with an ear tag for identification. Mice were anesthetized with CO₂ and intranasally inoculated with 50 μL of PBS (control group) or 50 μL of SIV suspension (EID₅₀ = 10^6^; infected group). Clinical signs were observed daily, and body weight was recorded at 9 a.m. for 14 days post-infection. At 72 h post-infection, three mice from each group were randomly selected and euthanized. The heart, liver, spleen, lungs, kidneys, and brain were collected and homogenized in PBS. Tissue homogenates were centrifuged at 3,000 × g for 10 min at 4 °C, and viral titers in the supernatants were determined as described in Section 2.4 to assess viral replication in each organ. Additionally, lung tissues were fixed in 4% paraformaldehyde, dehydrated, embedded in paraffin, sectioned, and stained with hematoxylin and eosin (HE) for histopathological examination under a light microscope.

## 3 Results

### 3.1 Virus isolation, identification, and whole-genome analysis

A total of 160 nasal swab samples were collected from pigs at a slaughterhouse in Qingdao, Shandong Province, China. The samples were first tested for HA activity, and one sample showed a positive HA titer of 8 log₂, which was also confirmed to contain SIV by RT-PCR, giving a positivity rate of 0.62%. An H1N2 virus was successfully isolated from this sample and named Sw/SD/QD726/2021 (H1N2) for further study. BLAST analysis of all eight gene segments revealed that PB2, PB1, PA, NP, M, and NS shared 98.33–99.13% sequence similarity with A/swine/Hong Kong/702/2015 (mixed), HA shared 98.71% similarity with A/swine/Liaoning/CY102/2014 (H1N1), and NA shared 95.96% similarity with A/swine/Henan/YIL/2010 (H1N2), respectively (Table 1). These results indicated that the isolate is closely related to the Hong Kong H1N2 mixed-subtype virus but contained gene segments from viruses circulating in other regions, suggesting that it may be a reassortant virus.

**Table 1:** Sequence homology of each gene segment of Sw/SD/QD726/2021 with the closest strains in NCBI.

Gene segment	Closest strain	Sequence identity (%)
PB2	A/swine/Hong Kong/702/2015 (mixed)	98.64
PB1	A/swine/Hong Kong/702/2015 (mixed)	98.86
PA	A/swine/Hong Kong/702/2015 (mixed)	98.70
HA	A/swine/Liaoning/CY102/2014 (H1N1)	98.71
NP	A/swine/Hong Kong/702/2015 (mixed)	99.13
NA	A/swine/Henan/YIL/2010 (H1N2)	95.96
M	A/swine/Hong Kong/702/2015 (mixed)	98.88
NS	A/swine/Hong Kong/702/2015 (mixed)	98.33

### 3.2 Geographic distribution and temporal characteristics of H1N2 SIV isolates

**Figure 1:**
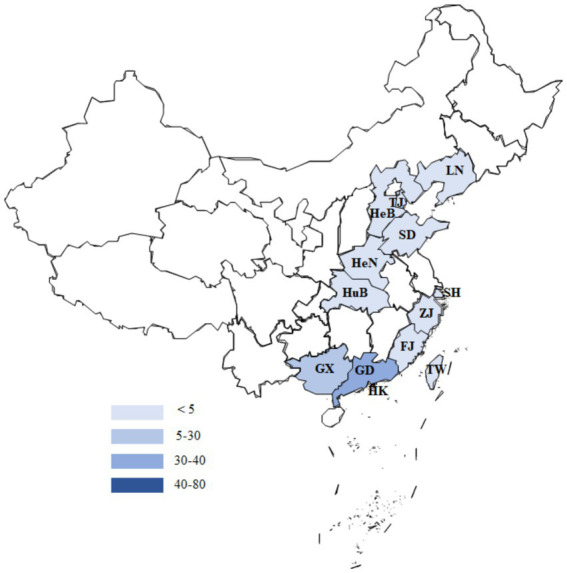
Geographic distribution of H1N2 SIV in China. The number of viral isolates in each province is shown, with increasing shades of blue indicating an increase in the number of H1N2 SIV isolates from 1 to 80.

**Figure 2:**
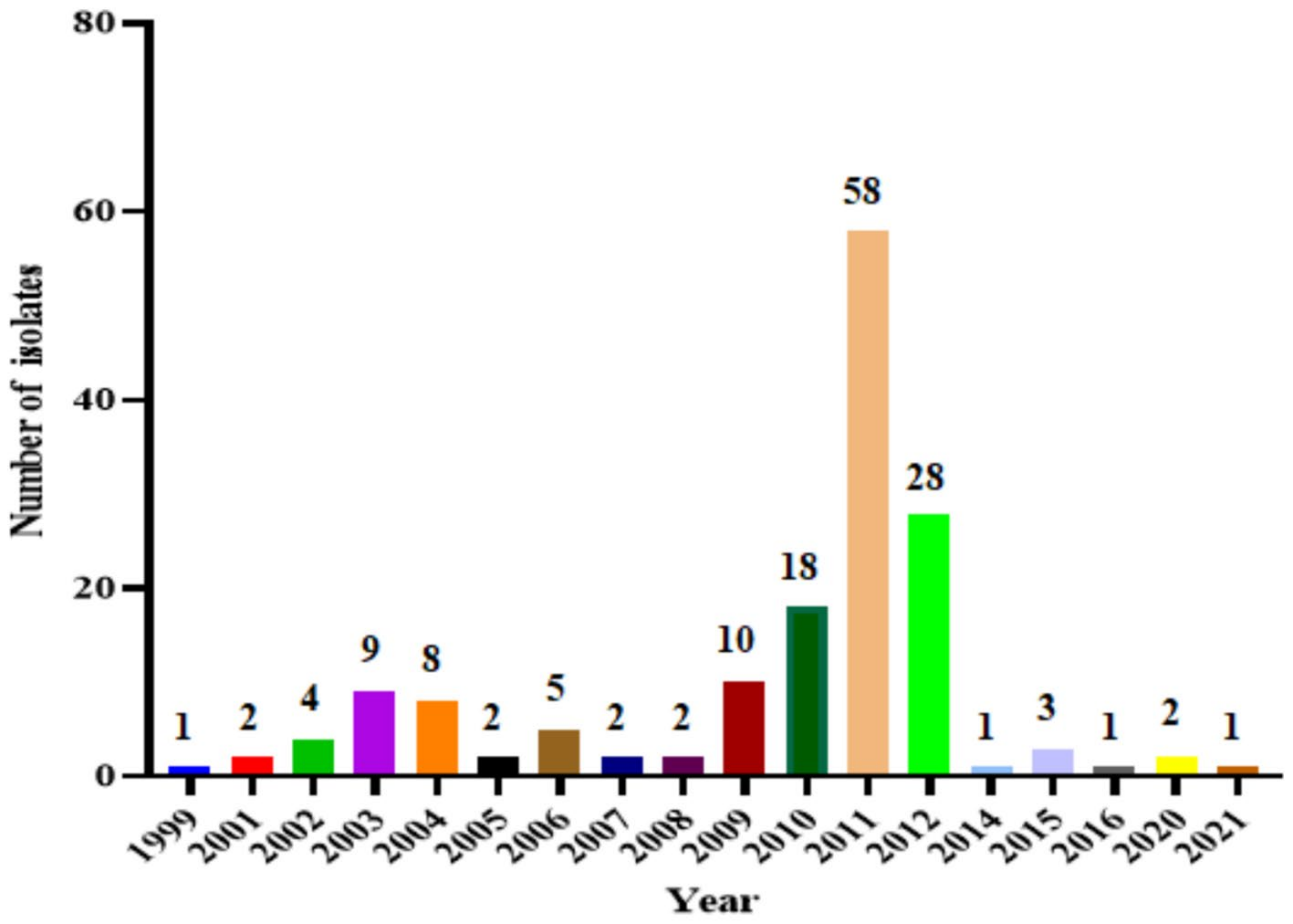
Annual distribution of H1N2 SIV between 1999 and 2021. The data reflect the number of reported isolates per year.

To systematically investigate the epidemiology of H1N2 SIV in China, we collected full-genome sequences of Chinese H1N2 SIV isolates from the GISAID database and the NCBI Influenza Virus Resource and analyzed their geographic and temporal distribution (Figures 1, 2). A total of H1N1 subtype SIV-positive samples were obtained from 11 provinces, 1 municipality, and 1 special administrative region. The spatial distribution of positive samples was uneven, with the highest rates in southern regions: Hong Kong (76 strains, 48.41%), Guangdong (33 strains, 21.02%), and Guangxi (28 strains, 17.83%). Other regions had fewer positive samples, including Zhejiang and Liaoning (4 strains each, 2.55%), Henan (3 strains, 1.91%), Tianjin and Hubei (2 strains each, 1.27%), and Shanghai, Fujian, Hebei, Shandong, and Taiwan (1 case each, 0.64%), suggesting higher viral exposure or more complete surveillance in southern China.

Over time, the number of H1N1 subtype SIV-positive samples showed clear fluctuations. Strains peaked in 2011 (58 strains, 36.94%) and remained relatively high from 2010 to 2012 (2010, 18 strains, 11.46%; 2012, 28 strains, 17.83%). In contrast, the numbers were generally low from 1999–2009 and 2014–2021, with most years reporting fewer than 10 strains, and no positive samples were detected in 2013 or from 2017 to 2019 (0.00%), indicating that the prevalence of H1N1 subtype SIV had cyclical and phase-specific patterns.

### 3.3 Phylogenetic analysis

**Figure 3:**
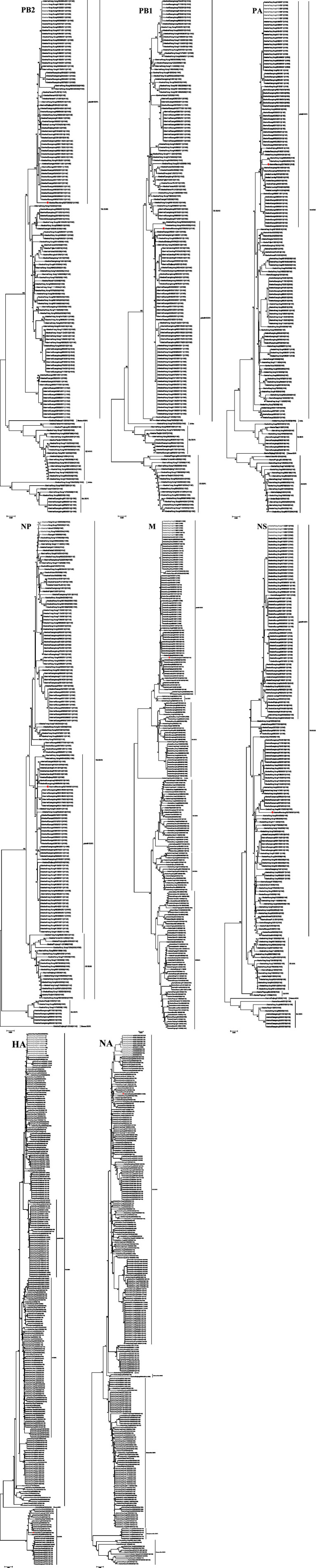
Phylogenetic trees of the PB2, PB1, PA, HA, NP, NA, M, and NS genes of 157 H1N2 SIV. The phylogenetic trees were constructed using the neighbor-joining method in MEGA 7.0, and the reliability of each tree was evaluated by 1,000 bootstrap replications. The isolates identified in this study are marked with solid red circles.

To better understand the genetic and evolutionary relationships of the H1N2 isolate, we constructed phylogenetic trees for eight gene segments using 156 reference sequences (Figure 3). The analysis showed that the HA gene of Sw/SD/QD726/2021 belongs to the EA H1N1 lineage, while its NA gene clusters with the TR H1N2 lineage, indicating that this strain may have acquired surface glycoproteins from different sources through reassortment. Further analysis of the internal genes revealed that PB2, PB1, PA, NP, and M segments originated from pdm/09 H1N1, whereas the NS gene was derived from TR H1N2. This combination of genes suggested that Sw/SD/QD726/2021 likely resulted from multiple reassortment events involving EA H1N1, TR H1N2, and pdm/09 H1N1 viruses, reflecting the complex evolution of H1N2 SIV in swine and potentially affecting its adaptation and pathogenicity.

### 3.4 Identification of a novel reassortant H1N2 SIV from pigs in Shandong, China

Representative H1N2 SIV isolates from different regions of China showed complex reassortment patterns, with gene segments coming from multiple lineages, including pdm/09 H1N1, EA H1N1, TR H1N2, CS H1N1, human-like H3N2, human H1N1, and European swine H1N1. Among the 28 genotypes identified (G1–G28), the internal genes (PB2, PB1, PA, NP, M, and NS) mainly came from TR H1N2 and pdm/09 H1N1, while HA and NA genes had diverse origins, including EA H1N1, CS H1N1, and TR H1N2.

Genotype G26 was the most common, with 36 strains (22.9%), followed by G8 and G21, each with 15 strains (9.6%). Other genotypes were less frequent, including G1 (13 strains, 8.3%), G2 (10 strains, 6.4%), G9 and G12 (9 strains each, 5.7%), G3 and G19 (7 strains each, 4.5%), and G20 (6 strains, 3.8%). Several genotypes (G4, G5, G6, G7, G13, G14, G15, G16, G17, G18, G22, G23, G24, G25, G27, G28) were rare, with only one or two strains each (0.6–1.3%) (Table 2).

**Table 2:** The genotypes of H1N2 SIV found in china from 1999–2021.

Strain	PB2	PB1	PA	HA	NP	NA	M	NS	Genotype	Number	Percentage (%)
A/swine/Hong Kong/NS728/2002(H1N2)									G1	13	8.28
A/swine/Shanghai/1/2007(H1N2)									G2	10	6.37
A/swine/Hong Kong/3125/2011(H1N2)									G3	7	4.46
A/swine/Henan/YIL/2010(H1N2)									G4	2	1.27
A/swine/Henan/4/2010(H1N2)									G5	1	0.64
A/swine/Tianjin/1/2007(H1N2)									G6	1	0.64
A/swine/Hong Kong/1479/2009(H1N2)									G7	1	0.64
A/swine/Hong Kong/1578/2003(H1N2)									G8	15	9.55
A/swine/Hong Kong/915/2004(H1N2)									G9	9	5.73
A/swine/Hong Kong/2314/2009(H1N2)									G10	5	3.18
A/swine/Guangxi/2887/2011(H1N2)									G11	5	3.18
A/swine/Guangxi/2910/2011(H1N2)									G12	9	5.73
A/swine/Hong Kong/5609/1999(H1N2)									G13	3	1.91
A/swine/Hong Kong/NS857/2001(H1N2)									G14	1	0.64
A/swine/Hong Kong/78/2003(H1N2)									G15	1	0.64
A/swine/Hong Kong/1304/2003(H1N2)									G16	1	0.64
A/swine/Hong Kong/M004/2020(H1N2)									G17	2	1.27
A/swine/Guangdong/3196/2011(H1N2)									G18	7	4.46
A/swine/Guangdong/617/2010(H1N2)									G19	6	3.82
A/swine/Guangdong/662/2012(H1N2)									G20	15	9.55
Sw/SD/QD726/2021(H1N2)									G21	1	0.64
A/swine/Hong Kong/263/2012(H1N2)									G22	1	0.64
A/swine/Hong Kong/NS584/2012(H1N2)									G23	1	0.64
A/swine/Hong Kong/715/2008(H1N2)									G24	1	0.64
A/swine/Guangxi/3202/2011(H1N2)									G25	1	0.64
A/swine/Hong Kong/4046/2011(H1N2)									G26	36	22.93
A/swine/Zhejiang/SW64/2014(H1N2)									G27	1	0.64
A/swine/Zhejiang/01/2008(H1N2)									G28	1	0.64

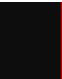
, Avian; 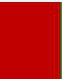, pdm/09 H1N1; 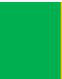, EA H1N1; 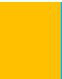, TR H1N2; 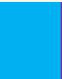, CS H1N1; 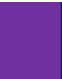, Human-like H3N2; 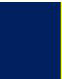, Human H1N1; 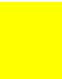, European swine H1N1.

Notably, the isolate Sw/SD/QD726/2021 belonged to a novel reassortant genotype, G21, characterized by a unique combination of all eight gene segments: PB2, PB1, PA, NP, and M from pdm/09 H1N1; HA from EA H1N1; and NA and NS from TR H1N2. This genotype was distinguished from previously reported genotypes by its specific combination of surface glycoproteins and internal genes, highlighting ongoing reassortment events in Chinese pig populations. The use of an eight-segment genotype framework ensures consistent classification and facilitates comparison with historical strains (Sun et al., 2025).

### 3.5 Analysis of key amino acid sites

In this study, we systematically analyzed key amino acid substitutions in Chinese H1N2 SIV strains and compared them with the isolated strain Sw/SD/QD726/2021 (Table 3). At position 627 of the PB2 protein, the majority of Chinese H1N2 SIV strains carried glutamic acid (E, 139 strains) or lysine (K, 18 strains), whereas Sw/SD/QD726/2021 retained E, a residue closely associated with polymerase activity, replication efficiency, and virulence in mammalian influenza viruses (Yamaji et al., 2015). At PB2 position 701, most strains carried aspartic acid (D, 147 strains) or asparagine (N, 10 strains), and Sw/SD/QD726/2021 retained D, which is critical for nuclear import and viral replication in mammalian hosts (Feng et al., 2015). For PB1, position 99 was predominantly histidine (H, 156 strains), with only one strain showing proline (P); Sw/SD/QD726/2021 retained H, a residue linked to enhanced transmission in ferret models (Linster et al., 2014). At PA position 336, leucine (L, 128 strains) was the major residue, whereas methionine (M) was found in 29 strains; Sw/SD/QD726/2021 retained L, which has been associated with increased polymerase activity in mouse models (Bussey et al., 2011).

**Table 3:** Molecular analysis of the Sw/SD/QD726/2021 virus.

Protein	Position	Mutations		Sw/SD/QD726/2021
PB2	627	E (139)	K (18)	E
	701	D (147)	N (10)	D
PB1	99	H (156)	P (1), Y (0)	H
PA	336	L (128)	M (29)	L
HA				
(H3 numbering)	Cleavage site	PSIQSR/GL (147)	PSIQTR/GL (10)	PSIQSR/GL
	190	E (7)	D (142), N (7), V (1)	N
	225	G (4)	D (122), E (26), G (4), N (4)	E
	226	Q (157)	L (0)	Q
	228	G (157)	S (0)	G
NA				
(N2 numbering)	119	E (157)	V (0)	E
	222	I (157)	L (0)	I
	224	R (157)	K (0)	R
	292	R (157)	K (0)	R
M2	31	S (40)	N (117)	N
NS1	42	P (0)	A (2), S (155)	S
	92	D (152)	E (5)	D

Mutations represent amino acid variations among 157 H1N2 SIV strains. Numbers in parentheses indicate strain counts.

Analysis of the hemagglutinin (HA, H3 numbering) cleavage site revealed that 10 strains carried PSIQTR/GL, while 147 strains carried PSIQSR/GL. Sw/SD/QD726/2021 harbored PSIQTR/GL, consistent with a low-pathogenicity influenza virus phenotype. Key HA residues involved in human-type receptor binding (positions 190, 225, 226, and 228) in Sw/SD/QD726/2021 were E, E, Q, and G, respectively (Matrosovich et al., 2000; Stevens et al., 2006). Specifically, position 190 in Chinese H1N2 SIV strains was predominantly D (142 strains), followed by E (7 strains), N (7 strains), and V (1 strain); position 225 was mainly D (122 strains), followed by E (26 strains), G (4 strains), and N (4 strains); positions 226 and 228 were fully conserved as Q and G in all 156 strains. These newly identified amino acid substitutions may have influenced viral binding to sialic acid receptors and warrant further functional evaluation.

In neuraminidase (NA, N2 numbering), position 119 was predominantly E, which Sw/SD/QD726/2021 retained; this residue is associated with resistance to oseltamivir and zanamivir (Zhao et al., 2024). Mutations at positions 222, 224, and 292 in NA were rare. At M2 position 31, Sw/SD/QD726/2021 carried N, a substitution linked to amantadine resistance (Zhao et al., 2024). Analysis of nonstructural protein 1 (NS1) showed that position 42 in 155 strains exhibited a proline (P) to serine (S) substitution, whereas Sw/SD/QD726/2021 contained S, which may affect virulence in mouse models (Jiao et al., 2008). At position 92, D-to-E substitutions were observed in five strains, while Sw/SD/QD726/2021 retained D, a residue potentially influencing host antiviral responses (Li and Wang, 2007).

### 3.6 Pathogenicity of Sw/SD/QD726/2021 in mice

**Figure 4:**
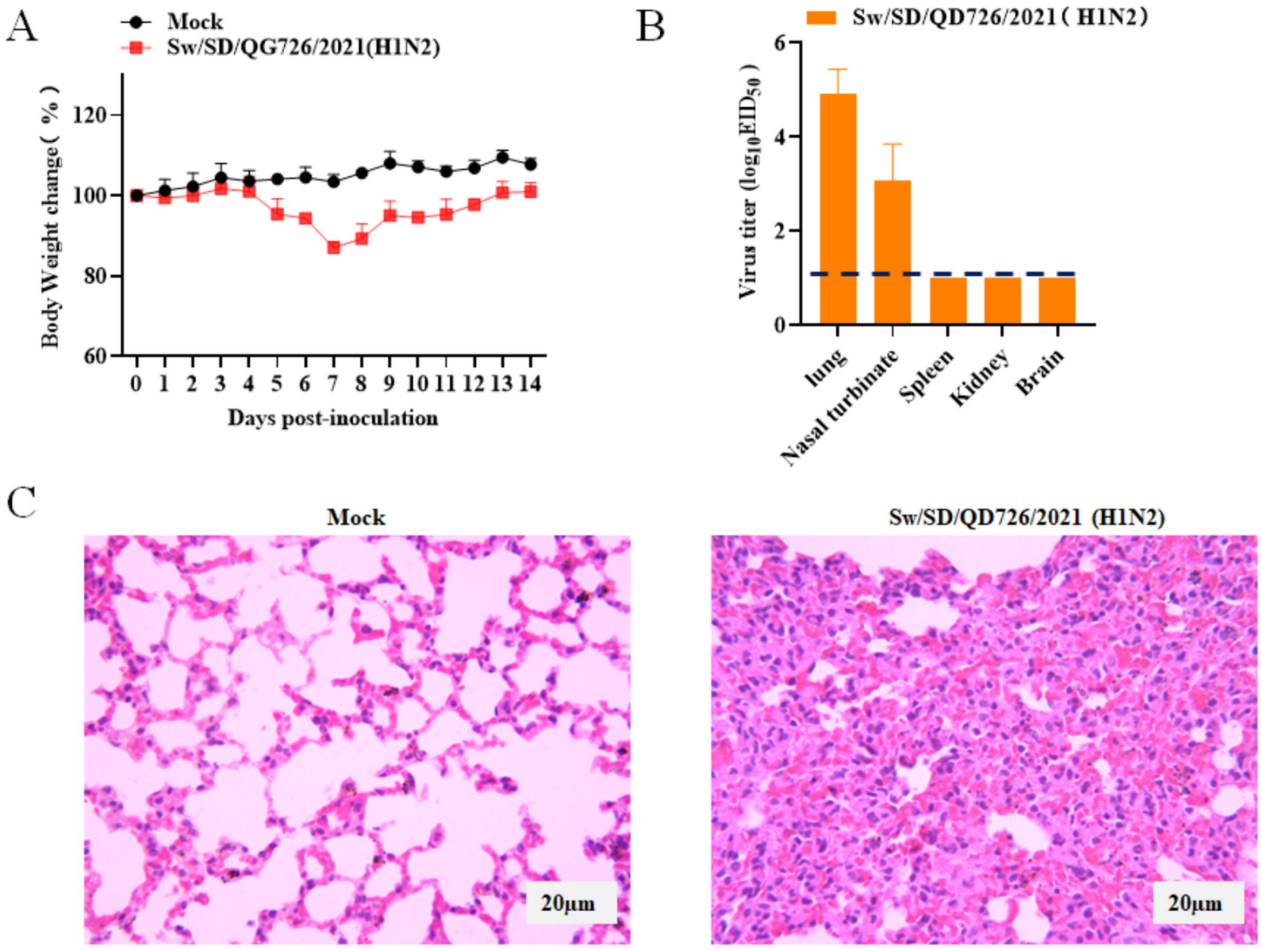
Body weight changes, viral replication, and lung histopathology in mice infected with Sw/SD/QD726/2021. Mice were intranasally inoculated with 10^6^ EID_50_ of virus in 50 μL. **(A)** Body weight changes are shown as the percentage relative to day 0 and were recorded daily for 14 days post-inoculation (*n* = 3). **(B)** Viral titers in different organs at 3 days post-inoculation, measured as log₁₀ EID_50_/mL. The dashed line indicates the detection limit. **(C)** Lung tissues were collected at 3 days post-inoculation, fixed in 10% formalin, embedded in paraffin, sectioned, and stained with HE. Histopathological changes were examined under a microscope at 400× magnification.

To evaluate the pathogenicity of the isolated strain Sw/SD/QD726/2021, six-week-old female BALB/c mice were intranasally inoculated with 10^6^ EID₅₀ of the virus, while the control group received an equal volume of PBS. As shown in Figure 4A, the body weight of infected mice decreased noticeably after infection, reaching the maximum loss of 13.02% on day 7 post-inoculation, followed by gradual recovery. In contrast, no significant change was observed in the control group. Virus titration revealed that the strain replicated efficiently in the respiratory tract of mice. The mean viral titers were 4.75 log₁₀EID₅₀/mL in the lungs and 3.08 log₁₀EID₅₀/mL in the nasal turbinates. No virus was detected in the spleen, kidneys, or brain (Figure 4B). Histopathological examination demonstrated that the lungs of infected mice exhibited marked thickening of the alveolar walls, inflammatory cell infiltration, and partial destruction of the alveolar structure, whereas the control group showed normal pulmonary architecture (Figure 4C). Collectively, these results indicated that Sw/SD/QD726/2021 replicated efficiently in the respiratory tract of mice and induced moderate pathological lesions.

## 4 Discussion

Pigs play an important role in the transmission of influenza viruses and the generation of triple-reassortant viruses because they are highly susceptible to both avian and mammalian influenza viruses. This susceptibility allows different influenza viruses to reassort in pigs, which contributes to viral evolution (Obadan et al., 2015). In this study, we performed whole-genome sequencing of a newly isolated H1N2 SIV, Sw/SD/QD726/2021, and compared each gene segment with reference sequences from the NCBI database to investigate its possible origin. The results showed that multiple gene segments of Sw/SD/QD726/2021 were highly similar to strains isolated in 2010, 2014, and 2015 from Liaoning, Henan, and Hong Kong, suggesting that this virus may have arisen from reassortment events involving these strains. Since the first isolation of H1N2 SIV in China, 28 different genotypes were identified. Following the introduction of the 2009/H1N1 virus into pigs, the frequency of reassortant viruses increased and the patterns of reassortment became more diverse. Whether the H1N2 genotype identified in this study had adapted to pigs and gained a certain epidemiological advantage requires continuous surveillance.

Regarding key viral proteins, residue 225E of the HA protein is important for airborne transmission of Eurasian avian-like H1N1 SIV in guinea pigs (Zhang et al., 2021). The Sw/SD/QD726/2021 strain carried 225E, and a new mutation at position 190 was also observed, which may affect receptor binding and needs further study. The M2 protein residue 31 N is associated with resistance to adamantane drugs, and the isolate in this study also carried this mutation. Among 156 H1N2 strains isolated in China, 117 carried the 31 N mutation. In addition, residue 42 of the NS1 protein, which is known to enhance virulence in mice, was present in Sw/SD/QD726/2021; notably, 155 of the 156 H1N2 strains analyzed also carried this mutation.

Mouse infection experiments showed that Sw/SD/QD726/2021 replicated efficiently in the lungs and nasal turbinates and caused significant weight loss, indicating moderate pathogenicity. These findings were consistent with previous reports (Yang et al., 2015; Lee et al., 2015). Other studies have shown that some influenza viruses can also replicate in the spleen and kidneys (Cui et al., 2024). However, mice are not the natural host for SIV and are not ideal for assessing interspecies transmission. Therefore, while these results indicate the virus can replicate in a mammalian host, they cannot directly predict its ability to transmit between pigs and humans. Future studies using pig models or ex vivo human airway tissues are needed to evaluate cross-species transmission potential.

From a public health perspective, H1N2 viruses are of interest because previous studies have reported occasional human infections and reassortment events. Our findings provided additional genetic and pathogenic data that may inform future surveillance. Since 2003, human-origin H1N2 and human–swine reassortant viruses in Canadian pigs have shown that human H1N2 viruses can infect pigs, and viruses carrying human PB1 and swine PB2/PA polymerase complexes can replicate in pigs (Karasin et al., 2006). During outbreaks of acute respiratory disease in Brazilian pig herds, human-like H1N2 viruses were also detected (Schaefer et al., 2015). A novel H1N2 reassortant virus, previously unreported in humans, was isolated from a swine farm worker with influenza-like illness in southeastern Brazil. This virus was a triple-reassortant, with HA from H1N2, NA from H3N2, and other internal genes from pandemic H1N1 (Resende et al., 2017). In 2021, Taiwan reported the first isolation of an H1N2v virus (A/Taiwan/1/2021(H1N2) v) from a 5-year-old girl, which resulted from reassortment between swine H1N2 and human pdm09/H1N1, showing that bidirectional transmission between pigs and humans increased influenza virus diversity and may posed a potential pandemic risk (Yang et al., 2022).

Bidirectional transmission allowed H1N2 viruses to circulate between hosts and provided opportunities for new reassortant viruses to emerge. Such reassortment may have changed viral pathogenicity and transmissibility and reduce the effectiveness of existing vaccines and antiviral drugs. For example, cases in Brazil and Taiwan showed that H1N2 can reassort with H3N2 or pdm09 H1N1 to form triple-reassortant viruses with genes from different sources, which lacked immunity in humans and posed potential pandemic risks. Moreover, bidirectional transmission makes virus monitoring and control more challenging. Pigs, as “mixing hosts,” may have generated new virus strains without being detected, while people in close contact with pigs, such as farm workers and children, are at higher risk of infection. If a new virus becomes highly transmissible, it could spread rapidly in communities or across regions, causing serious public health problems. Therefore, enhancing surveillance in pigs and high-risk groups, updating vaccines, and implementing protective measures are crucial for controlling H1N2 and its reassortants. In China, co-circulation of H1 and H3 subtypes in pigs provided the basis for the emergence of new reassortant viruses (Zhao et al., 2024; Sun et al., 2020), highlighting the importance of continuous monitoring and early warning of SIV to assess and reduce the risk of human infection.

## Data Availability

The original contributions presented in the study are included in the article/supplementary material, further inquiries can be directed to the corresponding authors.

## Footnotes

1
http://platform.gisaid.org/epi3/frontend

2
http://www.ncbi.nlm.nih.gov/genomes/FLU/FLU.html

## References

[ref1] Ali A. Khatri M. Wang L. Saif Y. M. Lee C. W. (2012). Identification of swine H1N2/pandemic H1N1 reassortant influenza virus in pigs, United States. Vet. Microbiol. 158, 60–68. doi: 10.1016/j.vetmic.2012.02.014, 22397932

[ref2] Bourret V. Lyall J. Frost S. D. W. Teillaud A. Smith C. A. Leclaire S. . (2017). Adaptation of avian influenza virus to a swine host. Virus Evol. 3:vex007. doi: 10.1093/ve/vex007, 28458917 PMC5399929

[ref3] Bussey K. A. Desmet E. A. Mattiacio J. L. Hamilton A. Bradel-Tretheway B. Bussey H. E. . (2011). PA residues in the 2009 H1N1 pandemic influenza virus enhance avian influenza virus polymerase activity in mammalian cells. J. Virol. 85, 7020–7028. doi: 10.1128/JVI.00522-11, 21561908 PMC3126589

[ref4] Charoenvisal N. Keawcharoen J. Sreta D. Chaiyawong S. Nonthabenjawan N. Tantawet S. . (2013). Genetic characterization of Thai swine influenza viruses after the introduction of pandemic H1N1 2009. Virus Genes 47, 75–85. doi: 10.1007/s11262-013-0927-x, 23740270

[ref5] Cui X. Ma J. Pang Z. Chi L. Mai C. Liu H. . (2024). The evolution, pathogenicity and transmissibility of quadruple reassortant H1N2 swine influenza virus in China: a potential threat to public health. Virol. Sin. 39, 205–217. doi: 10.1016/j.virs.2024.02.002, 38346538 PMC11074646

[ref6] Feng X. Wang Z. Shi J. Deng G. Kong H. Tao S. . (2015). Glycine at position 622 in PB1 contributes to the virulence of H5N1 avian influenza virus in mice. J. Virol. 90, 1872–1879. doi: 10.1128/JVI.02387-15, 26656683 PMC4733975

[ref7] Hoffmann E. Stech J. Guan Y. Webster R. G. Perez D. R. (2001). Universal primer set for the full-length amplification of all influenza A viruses. Arch. Virol. 146, 2275–2289. doi: 10.1007/s007050170002, 11811679

[ref8] Iwatsuki-Horimoto K. Nakajima N. Shibata M. Takahashi K. Sato Y. Kiso M. . (2017). The microminipig as an animal model for influenza A virus infection. J. Virol. 91:e01716. doi: 10.1128/JVI.01716-16, 27807225 PMC5215345

[ref9] Jiao P. Tian G. Li Y. Deng G. Jiang Y. Liu C. . (2008). A single-amino-acid substitution in the NS1 protein changes the pathogenicity of H5N1 avian influenza viruses in mice. J. Virol. 82, 1146–1154. doi: 10.1128/JVI.01698-07, 18032512 PMC2224464

[ref10] Joshi L. R. Knudsen D. Piñeyro P. Dhakal S. Renukaradhya G. J. Diel D. G. (2021). Protective efficacy of an Orf virus-vector encoding the hemagglutinin and the nucleoprotein of influenza A virus in swine. Front. Immunol. 12:747574. doi: 10.3389/fimmu.2021.747574, 34804030 PMC8602839

[ref11] Kanehira K. Takemae N. Uchida Y. Hikono H. Saito T. (2014). Reassortant swine influenza viruses isolated in Japan contain genes from pandemic A(H1N1) 2009. Microbiol. Immunol. 58, 327–341. doi: 10.1111/1348-0421.12152, 24750464

[ref12] Karasin A. I. Carman S. Olsen C. W. (2006). Identification of human H1N2 and human-swine reassortant H1N2 and H1N1 influenza A viruses among pigs in Ontario, Canada (2003 to 2005). J. Clin. Microbiol. 44, 1123–1126. doi: 10.1128/JCM.44.3.1123-1126.2006, 16517910 PMC1393092

[ref13] Lange J. Groth M. Schlegel M. Krumbholz A. Wieczorek K. Ulrich R. . (2013). Reassortants of the pandemic (H1N1) 2009 virus and establishment of a novel porcine H1N2 influenza virus, lineage in Germany. Vet. Microbiol. 167, 345–356. doi: 10.1016/j.vetmic.2013.09.024, 24139631

[ref14] Lee J. H. Pascua P. N. Decano A. G. Kim S. M. Park S. J. Kwon H. I. . (2015). Evaluation of the zoonotic potential of a novel reassortant H1N2 swine influenza virus with gene constellation derived from multiple viral sources. Infect. Genet. Evol. 34, 378–393. doi: 10.1016/j.meegid.2015.06.005, 26051886

[ref15] Li M. Wang B. (2007). Homology modeling and examination of the effect of the D92E mutation on the H5N1 nonstructural protein NS1 effector domain. J. Mol. Model. 13, 1237–1244. doi: 10.1007/s00894-007-0245-0, 17917748

[ref16] Linster M. van Boheemen S. de Graaf M. Schrauwen E. J. A. Lexmond P. Mänz B. . (2014). Identification, characterization, and natural selection of mutations driving airborne transmission of A/H5N1 virus. Cell 157, 329–339. doi: 10.1016/j.cell.2014.02.040, 24725402 PMC4003409

[ref17] Matrosovich M. Tuzikov A. Bovin N. Gambaryan A. Klimov A. Castrucci M. R. . (2000). Early alterations of the receptor-binding properties of H1, H2, and H3 avian influenza virus hemagglutinins after their introduction into mammals. J. Virol. 74, 8502–8512. doi: 10.1128/jvi.74.18.8502-8512.2000, 10954551 PMC116362

[ref18] Obadan A. O. Kimble B. J. Rajao D. Lager K. Santos J. J. S. Vincent A. . (2015). Replication and transmission of mammalian-adapted H9 subtype influenza virus in pigs and quail. J. Gen. Virol. 96, 2511–2521. doi: 10.1099/vir.0.000190, 25986634 PMC4635494

[ref19] Resende P. C. Born P. S. Matos A. R. Motta F. C. Caetano B. C. Debur M. D. . (2017). Whole-genome characterization of a novel human influenza A(H1N2) virus variant, Brazil. Emerg. Infect. Dis. 23, 152–154. doi: 10.3201/eid2301.161122, 27983507 PMC5176240

[ref20] Schaefer R. Rech R. R. Gava D. Cantão M. E. da Silva M. C. Silveira S. . (2015). A human-like H1N2 influenza virus detected during an outbreak of acute respiratory disease in swine in Brazil. Arch. Virol. 160, 29–38. doi: 10.1007/s00705-014-2223-z, 25209152

[ref21] Song Y. Wu X. Wang N. Ouyang G. Qu N. Cui J. . (2016). A novel H1N2 influenza virus related to the classical and human influenza viruses from pigs in Southern China. Front. Microbiol. 7:1068. doi: 10.3389/fmicb.2016.01068, 27458456 PMC4937032

[ref22] Starick E. Lange E. Grund C. Grosse Beilage E. Döhring S. Maas A. . (2012). Reassortants of pandemic influenza A virus H1N1/2009 and endemic porcine HxN2 viruses emerge in swine populations in Germany. J. Gen. Virol. 93, 1658–1663. doi: 10.1099/vir.0.042648-0, 22622326

[ref23] Stevens J. Blixt O. Glaser L. Taubenberger J. K. Palese P. Paulson J. C. . (2006). Glycan microarray analysis of the hemagglutinins from modern and pandemic influenza viruses reveals different receptor specificities. J. Mol. Biol. 355, 1143–1155. doi: 10.1016/j.jmb.2005.11.002, 16343533

[ref24] Sun H. Liu H. Zhang J. Qu X. Pang Z. Xu F. . (2025). Genome-scale evolution and phylodynamics of swine influenza A viruses in China: a genomic epidemiology study. Lancet Microbe 6:101020. doi: 10.1016/j.lanmic.2024.101020, 40311646

[ref25] Sun H. Xiao Y. Liu J. Wang D. Li F. Wang C. . (2020). Prevalent Eurasian avian-like H1N1 swine influenza virus with 2009 pandemic viral genes facilitating human infection. Proc. Natl. Acad. Sci. U.S.A. 117, 17204–17210. doi: 10.1073/pnas.1921186117, 32601207 PMC7382246

[ref26] Sun Y. Zhang J. Liu Z. Zhang Y. Huang K. (2021). Swine influenza virus infection decreases the protective immune responses of subunit vaccine against porcine circovirus type 2. Front. Microbiol. 12:807458. doi: 10.3389/fmicb.2021.807458 35003038 PMC8740023

[ref27] Trebbien R. Bragstad K. Larsen L. E. Nielsen J. Bøtner A. Heegaard P. M. . (2013). Genetic and biological characterisation of an avian-like H1N2 swine influenza virus generated by reassortment of circulating avian-like H1N1 and H3N2 subtypes in Denmark. Virol. J. 10:290. doi: 10.1186/1743-422X-10-290, 24047399 PMC3851529

[ref28] Yamaji R. Yamada S. Le M. Q. Ito M. Sakai-Tagawa Y. Kawaoka Y. (2015). Mammalian adaptive mutations of the PA protein of highly pathogenic avian H5N1 influenza virus. J. Virol. 89, 4117–4125. doi: 10.1128/JVI.03532-14, 25631084 PMC4442342

[ref29] Yang H. Chen Y. Qiao C. Xu C. Yan M. Xin X. . (2015). Two different genotypes of H1N2 swine influenza virus isolated in northern China and their pathogenicity in animals. Vet. Microbiol. 175, 224–231. doi: 10.1016/j.vetmic.2014.11.031, 25542286

[ref30] Yang J. R. Kuo C. Y. Yu I. L. Kung F. Y. Wu F. T. Lin J. S. . (2022). Human infection with a reassortant swine-origin influenza A(H1N2)v virus in Taiwan, 2021. Virol. J. 19:63. doi: 10.1186/s12985-022-01794-2, 35392932 PMC8988477

[ref31] Yu H. Zhang P. C. Zhou Y. J. Li G. X. Pan J. Yan L. P. . (2009). Isolation and genetic characterization of avian-like H1N1 and novel ressortant H1N2 influenza viruses from pigs in China. Biochem. Biophys. Res. Commun. 386, 278–283. doi: 10.1016/j.bbrc.2009.05.056, 19460353

[ref32] Zhang X. Li Y. Jin S. Zhang Y. Sun L. Hu X. . (2021). PB1 S524G mutation of wild bird-origin H3N8 influenza A virus enhances virulence and fitness for transmission in mammals. Emerg. Microbes Infect. 10, 1038–1051. doi: 10.1080/22221751.2021.1912644, 33840358 PMC8183522

[ref33] Zhao Y. Han L. Sang H. Yang P. Hou Y. Xiao Y. (2024). Two genotypes of H3N2 swine influenza viruses identified in pigs from Shandong Province, China. Front. Cell. Infect. Microbiol. 14:1517023. doi: 10.3389/fcimb.2024.1517023, 39748885 PMC11694508

